# The Controversial Role of Fibrosis in Autosomal Dominant Polycystic Kidney Disease

**DOI:** 10.3390/ijms21238936

**Published:** 2020-11-25

**Authors:** Maria Fragiadaki, Fiona M. Macleod, Albert C. M. Ong

**Affiliations:** Department of Infection, Immunity and Cardiovascular Disease, Faculty of Medicine, Dentistry & Health, University of Sheffield, Sheffield S10 2RX, UK; FMacleod1@sheffield.ac.uk (F.M.M.); a.ong@sheffield.ac.uk (A.C.M.O.)

**Keywords:** ADPKD, fibrosis, EMT, TGFβ, JAK/STAT, hypoxia, microRNAs, extracellular matrix (ECM), *PKD1*, *PKD2*

## Abstract

Autosomal Dominant Polycystic Kidney Disease (ADPKD) is characterized by the progressive growth of cysts but it is also accompanied by diffuse tissue scarring or fibrosis. A number of recent studies have been published in this area, yet the role of fibrosis in ADPKD remains controversial. Here, we will discuss the stages of fibrosis progression in ADPKD, and how these compare with other common kidney diseases. We will also provide a detailed overview of some key mechanistic pathways to fibrosis in the polycystic kidney. Specifically, the role of the ‘chronic hypoxia hypothesis’, persistent inflammation, Transforming Growth Factor beta (TGFβ), Janus Kinase/Signal Transducers and Activators of Transcription (JAK/STAT) and microRNAs will be examined. Evidence for and against a pathogenic role of extracellular matrix during ADPKD disease progression will be provided.

## 1. ADPKD

Autosomal dominant polycystic kidney disease (ADPKD) is the most common genetic renal disease worldwide. In Europe, patients with ADPKD account for ~10% of all end-stage renal disease (ESRD) requiring renal replacement therapy [1]. This disease is primarily caused by pathogenic mutations in the *PKD1* or *PKD2* genes. Phenotypically, ADPKD is characterized by progressive bilateral renal cyst growth, hypertrophy and fibrosis [2]. Progressive renal fibrosis is considered one of the hallmarks of ADPKD, which directly contributes to loss of renal function [3]. Currently, the only treatment available to delay disease progression is tolvaptan, which is contraindicated in some patients and can cause serious side effects, such as liver failure, in others [4].

### 1.1. ADPKD and Fibrosis

**Figure 1:**
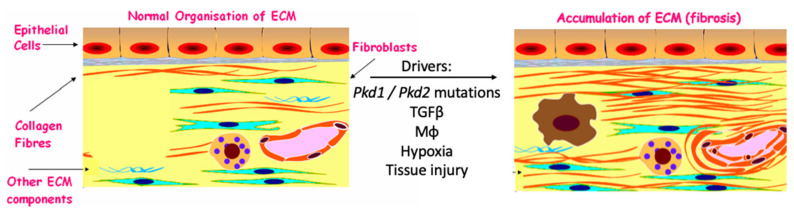
From normal to fibrotic interstitium. The ECM is a dynamic scaffold that is responsible for arranging cells and cell behaviour. Type I collagen is the most abundant collagen in our body. Under normal conditions a small number of collagen fibers is present in the kidney, but when the kidney is injured, there is an excessive accumulation of fibrillar collagens, contributing to tissue scarring. Some of the drivers of tissue injury and fibrosis in the polycystic kidney, such as macrophages (Mφ), will be described in more detail below.

Fibrosis is defined as the excessive accumulation of extracellular matrix proteins (ECM), which is a direct outcome of dysregulation of a number of processes (and related pathways) in response to tissue injury [5]. ECM is composed of a number of proteins, with type I collagen being the most abundant protein of our body [6]. Fibrosis progression is divided into three stages: (i) initiation, followed by (ii) persistent inflammation, and lastly (iii) established fibrosis. In humans, it is only in the embryo where tissue can be repaired without inflammation, scarring or fibrosis [7,8], with the embryonic mechanisms of scar-free healing remaining elusive. Established fibrosis is defined as tissue overgrowth, hardening and often scarring (Figure 1), characterized by excessive deposition of matrix proteins, such as collagen fibers. We will describe below the three stages of fibrogenesis and highlight original research that has shed light into the mechanisms responsible for these in the context of ADPKD.

### 1.2. Initiation Stage of Fibrosis

Whilst initiation of renal fibrosis in many renal pathologies is frequently due to renal parenchymal injury (e.g., ischemia, trauma, toxicity), in the case of ADPKD, the underlying cause of renal injury is genetic mutations hence has the property of being inherited from one generation to the next. A key contributory event in the initiation of fibrosis in ADPKD, is the inheritance of germline mutations in the *PKD1* [9,10,11,12] or *PKD2* [13] genes, which encode for the two polycystin proteins which co-localise in the primary cilia and other compartments [14] and interact with each other [15]. Yet the precise function(s) of the polycystins remain unknown. While mutations in the *PKD1* gene cause the majority of ADPKD cases (80%) [16], the remaining cases are due to mutations in *PKD2* and other ADPKD-causing genes. A few other genes have been reported to cause Autosomal Dominant Polycystic Kidney Disease in humans, including PKD3 [17]. To these genes, a few ‘modifier’ genes can be added. These genes are identified due to their capacity of either causing or enhancing progression of polycystic kidney disease. An example of these modifier genes is the RNA-binding protein bicaudal C, which via regulating PKD2, controls cyst formation [18]. The fact that ADPKD follows a dominant form of inheritance makes disease progression and fibrosis predictable aspects of this disease. Indeed, fibrosis in murine models of polycystic kidney disease is delayed by a few weeks from the onset of cysts, with fibrosis appearing after 4–6 weeks of cystic kidney disease [19], suggesting that fibrosis is a consequence of tissue injury in PKD models and not a triggering factor. This observation has been reported by a number of independent studies [20], with some studies showing that when fibrosis is established, cystic growth regresses [21]. Regression of cystic disease with the onset of fibrosis is interesting because it supports the paradigm that ECM production is triggered, at least initially, as a protective reparative mechanism. Interestingly fibrosis, at least in the case of the polycystic kidney, causes regression of cystic disease, therefore supporting a potentially reno-protective role. Yet it is well established that matrix production over prolonged time exacerbates disease by establishing tissue scarring, leading to further tissue damage and ultimately organ failure. So, it can be speculated that, at least during the initial stages of PKD, fibrosis is a protective mechanism that limits disease in the polycystic kidney.

### 1.3. Middle Stage–Persistent Inflammation

Persistent inflammation, which is a direct response to kidney injury, is characterised by infiltration of immune cells, expansion of resident cells, followed by production of cytokines and other bioactive compounds, ultimately leading to enhanced ECM production. Inflammatory cells such as macrophages (Mφ) have been reported in both human and experimental models of ADPKD. While Mφ can possess either protective or disease-causing roles in other pathologies (such as atherosclerosis, where they become lipid-loaded foam cells), in ADPKD, evidence suggests that Mφ are primarily associated with disease progression. Karihaloo et al. showed that depletion of Mφ from Pkd1^fl/fl^;Pkhd1-cre mice (by treatment with liposomal clodronate), resulted in a significantly lower cystic index, reduced proliferation of tubular epithelial cells, better preserved renal parenchyma and importantly improved renal function [22]. It was subsequently confirmed by an independent study that Mφ are present during disease progression in human polycystic kidneys (dominant and recessive forms) and their depletion reduces cystogenesis in cpk mice, a model with non-orthologous polycystic kidney disease [23,24,25,26]. A question remains what are the signals that attract inflammatory cells in the kidney in the presence of *Pkd1* mutations? Viau et al., suggested that within the primary cilia a protein complex composed of the ciliary kinase LKB1 and ciliopathy-related genes, such as *PKD1* and *NPHP1*, suppress expression of CCL2 (monocyte chemoattractant protein 1, MCP1) [27], thus limiting the recruitment of Mφ. Moreover, an additional explanation for the observed increase of Mφ in the polycystic kidneys could be due to tubular atrophy and accompanied cell proliferation, two processes often observed at this stage of disease. Specific to ADPKD is the ability of tubular epithelial cells to over-proliferate leading to the relentless growth of cysts and thus the overall size of the kidneys [20]. It is hard to discuss Mφ without also mentioning renal fibroblasts, as they share many common markers. At this stage of disease, the resident fibroblasts, who mostly reside in the kidney interstitium, and some are also responsible for making erythropoietin [28], become activated and turn into myofibroblasts. Activated myofibroblasts rapidly produce excess levels of ECM components particularly fibrillar collagens (type I and III), type IV collagen and non-collagenous proteins such as fibronectin and tenascin C. The origin of ‘activated’ myofibroblasts in the kidney and where they come from is reviewed elsewhere for other causes of renal fibrosis [29,30] and is currently unknown for ADPKD.

### 1.4. Late Stage–Established Fibrosis

**Figure 2:**
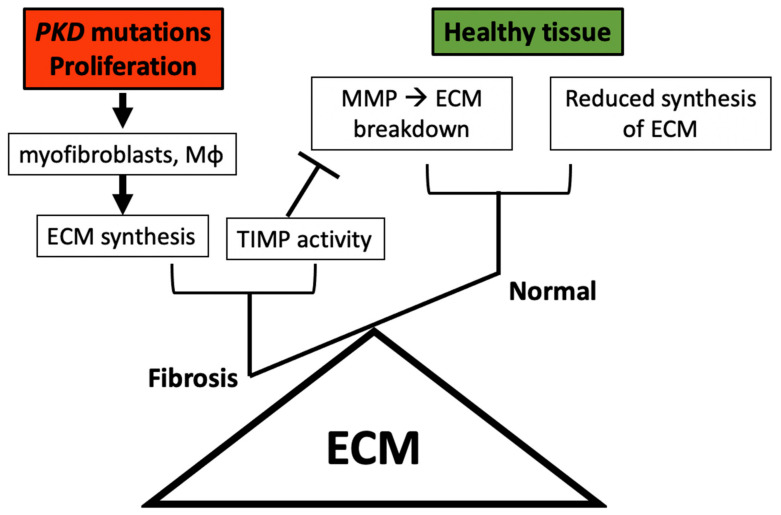
Tissue homeostasis requires a fine balance of matrix production and breakdown. Yet when the balance breaks, here due to somatic mutations in one of two genes (i.e., *PKD1* or *PKD2*, collectively referred to as *PKD*), there is an observed increased proliferation of matrix producing cells (e.g., myofibroblasts and Mφ), leading to excess ECM synthesis. To make matters worse in rodent models of *PKD* and also in people suffering with this condition, elevated levels of TIMPs have been identified. TIMPs inhibit the activity of the molecules that break down matrix (MMPs), further contributing to the establishment of fibrosis, which ultimately leads to organ failure. On the contrary, in the healthy kidney, there is both a lower rate of ECM production and also a tightly controlled ECM breakdown pathway.

Established fibrosis is defined by the presence of excess amounts of fibrous tissue which interferes with organ function. This can also be viewed as an imbalance in ECM deposition, which can be due to increased production (synthesis) or decreased break down (degradation) of matrix proteins. Matrix Metalloproteinases (MMPs) are a family of enzymes capable of matrix breakdown, including collagenases, gelatinases and stromelysins. Interestingly, and in contrast to the fact that ADPKD kidneys develop fibrosis, there are reports of elevated MMP1 activity in ADPKD [31], with increased MMP1 activity correlating with common ADPKD cardiovascular events (such as intracranial aneurysms, which is one of the most serious complications of ADPKD and often results in sudden death). On the contrary, Schaefer et al., reported that MMP2 (i.e., gelatinase A) is reduced in some models of PKD [32], while MMP-9 and MMP-3 are not expressed. Since MMPs are inhibited by Tissue inhibitor of metalloproteinases (TIMPs), it follows that enhanced TIMP activity will limit the action of the ECM breakdown, therefore contribute to fibrosis. Schaefer et al. further showed that the reduced renal tubular epithelial MMP2 activity was concurrent with an increase in TIMP-1 and TIMP-2 activity [32]. Nakamura et al. confirmed that TIMP-1 is elevated in patients with ADPKD when compared to healthy controls, making TIMP-1 a potentially patient-relevant ECM modulator [33]. A recent study of 296 patients reported a reduction in urinary TIMP-2 levels in ADPKD patients when compared to healthy controls [34], which suggests there is regulation taking place at the level of family subunits in the polycystic kidney. Taken together, more work needs to be carried out to define the precise mechanisms of matrix accumulation and breakdown in ADPKD. It is well known that for tissue homeostasis equilibrium to exist, a balance between matrix production, matrix turnover and TIMP activity must be achieved (Figure 2). If more matrix is made than required, this leads to the establishment of fibrosis, and ultimately organ failure. On the other hand, if too much of the body’s matrix is broken down, tissue degeneration, such as this seen in arthritis, takes place. Taken together, this review highlights that ECM turnover is abnormal in the polycystic kidney.

## 2. The Molecular Pathways that Control Fibrosis in ADPKD

A number of pathways contribute to ADPKD disease progression, some of which will be discussed below.

### 2.1. Chronic Hypoxia Hypothesis

ADPKD is a disease that is triggered by *PKD* mutations, but for this disease to fully develop a ‘second hit’, and sometimes a ‘third hit’, has to take place. One of those ‘hits’ could be hypoxia. It has been proposed that chronic hypoxia can contribute to tissue fibrosis in a number of renal diseases. The ‘chronic hypoxia hypothesis’ was first introduced in 1998 [35] where it was proposed that tissue hypoxia is a major contributor to tubulointerstitial fibrosis via induction of apoptosis and Epithelial to Mesenchymal Transdifferentiation (EMT, more on this below). Hypoxia-triggered apoptosis and EMT exacerbate fibrosis leading to a vicious cycle resulting in renal failure. Chronic hypoxia in the ADPKD kidney can be triggered either via reduced glomerular function, vasoconstriction and/or a reduction in the number of efferent arterioles. Indeed, a number of studies have suggested that glomerular damage is rare but can be observed in polycystic kidney disease, manifesting as podocyte injury [36] or glomerular cysts [37]. Glomerular injury is not surprising, given that *PKD1* is expressed within the glomerulus as well as in afferent arterioles [38]. Nie and Arend showed that deletion of *Pkd1* in mice led to a notable decrease of detectable vasculature [39]. On the other hand, microvascular damage has been shown to create a situation where the cells have limited oxygen supply, which in turn leads to a fibrotic response in tubulointerstitial cells [40,41]. This hypothesis is supported by experiments in *Pkd1* heterozygous mice which develop more severe renal cystic disease after ischemia reperfusion injury [42], suggesting that *PKD1* mutations increase the hypoxia sensitivity of the kidney. Hypoxia inducible factor 1 alpha (HIF1α) is a *bona fide* transcription factor induced by hypoxia and hence a marker of hypoxia [43]. The polycystic kidney has been shown to express HIF1α in tubular epithelial cysts, providing evidence that it experiences hypoxia [44,45,46]. Interestingly, *Pkd1* knockout mice treated with a HIF-prolyl hydroxylase (PHD) inhibitor, which activates HIF1α by limiting its PHD-dependent hydroxylation [47] and subsequent recognition by the proteome [48], displayed exacerbated cystic disease. The authors also showed that genetic deletion of the *HIF1α* gene reduced cystic growth, suggesting *HIF1α*, and by approximation also hypoxia, exacerbates cystic disease [44]. Interestingly, it has been shown that HIF1α protein can activate Ca^2+^-activated Cl- secretion via TMEM16A (anoctamin 1, ANO1) [44,49], while it was recently shown that TMEM16A significantly exacerbates cystic disease in vitro [49,50] and Ca^2+^ [51]. These findings provide further support that HIF1α and hypoxia promote disease in the polycystic kidney. Taken together, the polycystic kidney experiences increased levels of tissue hypoxia, while the proposed HIF1α, ΤΜΕΜ16A, ion secretion axis, which is shown to exacerbate disease, may become a successful target for therapy. Hence, genetic or pharmacologic means to suppress the effects of hypoxia and/or HIF1α actions, are predicted to have therapeutic benefit. Small molecule HIF inhibitors, which are actively studied as anticancer drugs, may prove to be of therapeutic benefit in ADPKD. It should be noted, however, that the use of HIF inhibitors may potentially be limited in ADPKD as HIFs are also essential for making erythropoietin, which in turn instructs an increase of red blood cells, via activating the Janus Kinase and Signal Transducers and Activators of Transcription cascade (more on JAK/STAT below).

### 2.2. Signalling Pathways Leading to Fibrosis in ADPKD

**Figure 3:**
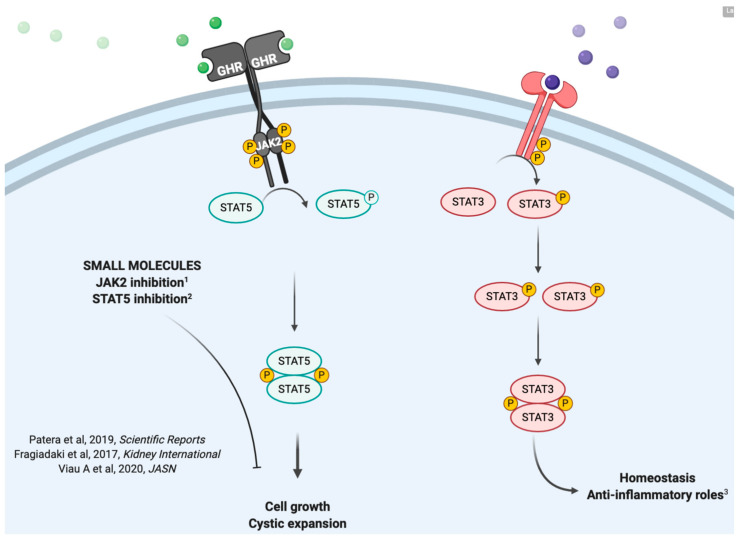
The role of JAK/STAT in cystic disease. The JAK/STAT family of evolutionarily conserved Janus Kinase and Signal Transducers and Activators of Transcription plays a key role in driving cystic growth in ADPKD. Specifically, JAK2-triggered STAT5 enhances cystic growth [20], and consistent with this, inhibition of JAK2 limits this [58]. On the other hand, STAT3, which can also be activated by JAK2, provides a homeostatic control, that when switched off leads to excessive renal inflammation [57].

A critical signalling pathway involved in EMT, fibrogenesis and cell proliferation (all processes associated with disease progression) is the Janus Kinase and Signal Transducers of Transcription (JAK/STAT). The JAK/STAT family is composed of 4 tyrosine kinases (namely JAK1-3 and Tyk2) and 7 transcription factors (STAT1-5a, STAT5b and STAT6). Altered activity of a number of JAK/STAT pathway components is identified in ADPKD and is proposed to be a defining feature of disease development [20,52,53]. Yet whether JAK/STAT controls fibrosis in the polycystic kidney is currently unknown. STAT5, drives proliferation in ADPKD while its inhibition, with siRNA or a small molecule inhibitor, blocks cell growth in human cellular models of ADPKD [20]. STAT5 has been recently found to co-operate with insulin-like growth factor 1 (IGF-1) to enhance EMT in hepatocellular carcinoma [54]. Specifically, STAT5 upregulates the expression of vimentin, *N*-cadherin, Snail1/2 and Twist-1, which are markers of EMT. Likewise, STAT3 is also involved in promoting EMT in cancer models [55]. Interestingly STAT3 was originally proposed to be a transcription factor that drives Polycystic Kidney Disease, as its expression is significantly elevated in models of ADPKD [56]. Yet it was recently conclusively shown that the action of STAT3 is to limit renal inflammation [57]. Hence STAT3 does not enhance cystic disease, instead STAT3 is a homeostatic transcription factor required for normal kidney function. JAK2 on the other hand, is a kinase that can activate both STAT3- (protective) and STAT5- (potentially pathogenic) triggered transcriptional programmes. JAK2 was shown to be highly elevated in mouse models of ADPKD and its inhibition blocked cystic growth [58], suggesting that is may be a promising therapeutic target. The relative contributions of JAK2, STAT5 and STAT3 in the progression of ADPKD are summarised in Figure 3. Taken together, it appears that JAK/STAT has a clear role in ADPKD disease development, yet its role in controlling potentially disease-causing processes such as EMT and fibrosis, as well as the precise molecular mechanisms underpinning these actions, require additional work in ADPKD.

## 3. Epithelial to Mesenchymal Transition (EMT) and the Polycystic Kidney

EMT is the process where epithelial cells may transdifferentiate into fibroblasts which become activated fibroblasts, also known as ‘myofibroblasts’. EMT-derived myofibroblasts have increased capacity to lay down matrix [59] and contribute to fibrosis progression. EMT is a key player in many chronic kidney diseases [29,60,61]; yet its role in the polycystic kidney disease is less well defined. A immunohistochemical study of a small number of human ADPKD kidneys showed increased expression of two classic EMT markers (i.e., α-SMA and vimentin) and decreased expression of epithelial markers (e.g., *E*-cadherin) [62]. Even though the later study is observational, it offers support for the possibility of EMT taking place in human ADPKD kidneys. Increased mesenchymal marker expression with a decrease in epithelial cell origin markers was also observed in an in vitro study, suggesting that EMT could contribute to ADPKD progression by enhancing cell migration [63]. However, it should be noted that in vitro studies of EMT are less reliable as cells ex vivo are more likely to undergo EMT compared to their in vivo counterparts [29]. Likewise, an in vivo study suggested that cyst lining cells acquire mesenchymal features in response to cyst enlargement and this event exacerbates fibrosis progression [64]. Taken together there is circumstantial evidence to support that EMT may be a feature of the polycystic kidney, though more research is required to address this topic, including lineage tracing coupled with high-resolution imaging.

**Figure 4:**
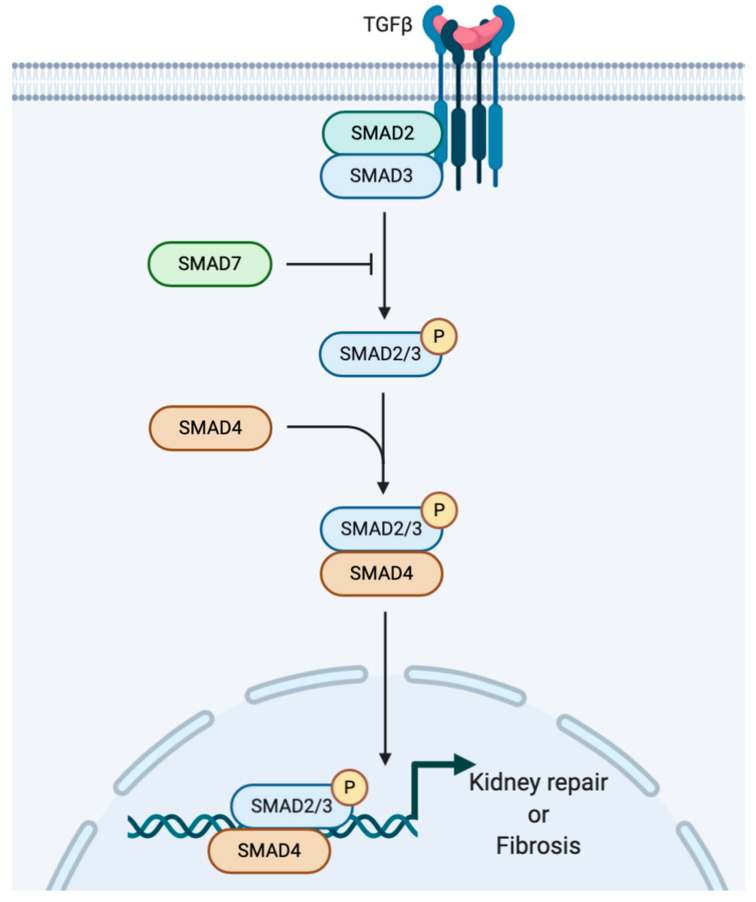
The role of TGFβ in renal fibrosis. TGFβ engages the ALK receptors to activate SMAD2-4 transcriptional programme. Initially TGFβ plays a protective role by enhancing ECM production, such as *COL1A2*, leading to tissue repair. However, excessive TGFβ signalling can lead to tissue scarring further complicating renal disease.

EMT is regulated by numerous growth factors, cytokines and hormones. TGFβ is one of the most important profibrotic factors in the kidney since it can not only initiate but can also maintain EMT [65]. Importantly, the actions of TGFβ can be counteracted by Bone Morphogenic Protein-7 (BMP-7), which is highly expressed in the kidney [66]. In mammals, the TGFβ family consists of 3 members (TGF-β1, TGF-β2 and TGF-β3), which have similar biological effects. The 3 cytokines belong to a larger family, the TGFβ superfamily, which also contains proteins such as bone morphogenetic proteins (BMP) [67]. TGFβ binds to TGFβ receptor 1 and 2 and triggers a signalling pathway that involves phosphorylation and transcriptional activation of SMAD2/3/4 transcription factors (Figure 4). TGFβ has been reported by several investigators to promote wound healing, tissue repair and regeneration after initial insult, such as inflammation as well as tissue fibrosis [68,69,70], and to be anti-inflammatory [71]. However, recent studies have uncovered a pro-inflammatory role for TGFβ in the development of plaque and atherosclerosis [72], suggesting that the role of this important transcription factor is complex and possibly tissue and trigger specific. TGFβ, apart from its roles in immune regulation, is a well-studied profibrotic molecule involved in progressing renal fibrosis by orchestrating a crosstalk between parenchymal, inflammatory and collagen expressing cells (e.g., fibroblasts). It is mainly produced by both resident renal cells and infiltrating macrophages but it is also made by monocytes, lymphocytes, platelets, fibroblasts and osteoblasts.

Increased TGFβ signalling has been reported in several animal models of ADPKD [73,74,75]. In contrast to its profibrotic disease promoting role in other disease models, Elberg et al., surprisingly found that cystic epithelial cells produce TGFβ2, which inhibits cystic disease in three-dimensional in vitro cyst assays [76], via an undefined mechanism. Further support for a protective effect of TGFβ2 comes from in vivo genetic deletion studies of ALK5, a cellular receptor for TGFβ, which did not adversely affect cystic disease in *Pkd1* knockout mice [77]. On the contrary it was recently shown that ALK5 may hold some therapeutic benefit, since small molecule ALK5 inhibitors were capable to suppress the curly tail phenotype in *Pkd2* deficient zebrafish [75]. These results need to be confirmed in other models, including mammalian systems. Taken together, the role of TGFβ signalling and whether it contributes to fibrosis and conversely disease progression or protection remains controversial in the ADPKD field and requires further study.

## 4. MicroRNAs

MicroRNAs (miRNAs) are a class of short non-coding RNAs (~22 nucleotides) that inhibit post-transcriptional gene expression through binding target sites in mRNA, usually in the 3′UTR region [78]. MicroRNAs play an important role in normal kidney development as regulators of cell survival, proliferation and apoptosis in nephron progenitors [79,80,81], exemplified in deletion of the miR-17~92 cluster in nephron progenitors which leads to impaired proliferation and nephrogenesis [82]. The importance of miRNAs in renal homeostasis has been demonstrated through deletion of Dicer, an enzyme required for miRNA biogenesis, which leads to cytoskeletal disruption and glomerular disease in podocyte-specific deletion [83], as well as lower numbers of juxtaglomerular cells and reduced expression of renin genes (*Ren1* and *Ren2*) with subsequent renal vascular abnormalities and fibrosis [84]. When aberrantly expressed, miRNAs have been implicated in the pathogenesis of ADPKD [85].

### 4.1. microRNAs as Biomarkers of Disease

Disease progression in ADPKD can be highly variable between patients which can make the age of onset of ESRD difficult to predict [86,87]. Dysregulated microRNAs have been analysed as potential biomarkers of disease progression. Analysis of miRNA profiles in urinary cells between 20 patients with ADPKD and 20 patients with chronic kidney disease (CKD) of other aetiologies revealed an increased abundance of miR-143, miR-223, miR-199a and miR-199b, with reduction in miR-133 and miR-1. Of note, miR-199a is associated with fibroblast activation and renal fibrosis [88], but its role in controlling fibrosis in ADPKD is currently unexplored. When miRNAs in urine microvesicles of patients with ADPKD were compared to patients with other causes of CKD, miR-1 and miR-133a were reduced in ADPKD [89]. Recently, it was reported that there is significant downregulation of kidney-enriched miR-192-5p, miR-194-5p, and members of the miR-30 family in urinary exosomes of patients with ADPKD as well as in kidneys of a murine PKD model and in vitro human cystic cell lines compared to healthy controls [90]. Members of the miR-192 family, miR-192 and miR-194, had also been reported to be reduced in PKD in intermediate and late stage cystogenesis. These two miRNAs can directly bind and suppress *ZEB2* and *CDH2* (i.e., N-cadherin) which are highly associated with epithelial-mesenchymal transition [91].

### 4.2. Disease-Altering microRNAs

#### 4.2.1. miR-17~92

The miR-17~92 cluster (comprised of miR-17, miR-18a, miR-19a, miR-20a, miR-19b-1 and miR-92a-1) is important in kidney development [82], is upregulated in many cancers and both directly and indirectly inhibits tumour suppressor genes [92]. In the context of ADPKD, the miR-17-92 cluster was shown to be upregulated in PKD models [93] and deletion of miR-17~92 leads to reduced interstitial fibrosis and cyst epithelial proliferation [94]. Treatment in murine preclinical models with anti-miR-17 reduced total kidney volume and cystic index as well as reduced expression of markers of fibrosis, *ACTA2* (i.e., α-SMA) and *CO1A1* (i.e., collagen I alpha 1) [94,95,96]. Taken together, suppression of the miR-17~92 cluster attenuates ADPKD progression including fibrosis in several mouse models of polycystic kidney disease.

#### 4.2.2. miR-21

Although miR-21 is not essential for nephrogenesis [97], it plays a role in tissue regeneration after injury by indirectly stimulating proliferation and inhibiting apoptosis. When persistently elevated, miR-21 promotes renal fibrosis through modulation of several metabolic pathways [98,99,100,101]. Interestingly, a number of cell signalling pathways that have been implicated in PKD pathogenesis also modulate or are modulated by miR-21 including JAK/STAT, cAMP-CREB, MAPK-ERK, and TGFβ-Smad pathways [100,102,103,104,105]. Aberrant upregulation of miR-21 has been observed in preclinical models of PKD, whereby there was a significant correlation between cystic disease and miR-21 expression. Upregulated miR-21 was also observed in renal tubules of patients with ADPKD [105]. Although overexpression of miR-21 alone is not sufficient to produce kidney cysts [97], miR-21 deletion in a PKD mouse model reduced cyst growth [105]. Anti-miR-21 RG-012 is currently in clinical trials for treatment of Alport syndrome but has not yet been tested in ADPKD. Taken together miR-21 is a promising microRNA, which could potentially be targeted therapeutically (use of RG-012), to test its effects in ADPKD.

In summary, microRNAs may provide novel strategies for determining prognosis and delaying progression of ADPKD, demonstrated by their role in cyst growth and fibrosis and by the use of effective novel nucleic-acid based treatments.

## 5. Summary and Concluding Comments

In conclusion, while excessive accumulation of ECM components and concomitant fibrosis is generally considered a major disease driver in many renal conditions, the role of fibrosis in ADPKD is controversial and requires further investigation. In the polycystic kidney, this fibrogenic response is generally correlated with cystic disease regression. How tissue fibrosis plays protective roles in the polycystic kidney is currently unclear and has not been directly addressed in the ADPKD field. Indirect evidence that fibrosis may protect the polycystic kidney comes from cell signalling studies, where master regulators of the fibrogenesis processes, such as TGFβ, have either protective or neutral roles in the development of cystic disease, suggesting that fibrosis in ADPKD may have some protective roles. Chronic hypoxia worsens cystic kidney disease, and this could be due to accumulation of ECM, or simply reflecting the disturbed movement of blood into and out of the kidney (altered blood flow). In contrast to TGFβ driven ECM production, which appears to be protective, macrophages also contribute to ECM production but are detrimental, arguing for a context-specific, complex, role of ECM in polycystic kidney disease. The JAK/STAT pathway appears to mediate important roles in controlling the growth of cysts, yet further work is needed to shed light into the precise molecular mechanisms, with an emphasis on cytokines/pathways that activate disease processes. While microRNAs have clearly been shown to control important processes associated with fibrogenesis outside of ADPKD, in the context of ADPKD their role in controlling fibrosis is an active and promising area of research. Taken together, fibrosis in ADPKD remains a controversial subject and substantive further mechanistic work is needed to determine its contribution to disease development.
